# Delay aversion but preference for large and rare rewards in two choice tasks: implications for the measurement of self-control parameters

**DOI:** 10.1186/1471-2202-7-52

**Published:** 2006-06-23

**Authors:** Walter Adriani, Giovanni Laviola

**Affiliations:** 1 Behavioural Neuroscience Section, Dept. Cell Biology & Neurosciences, Istituto Superiore di Sanità, Roma, Italy

## Abstract

**Background:**

Impulsivity is defined as intolerance/aversion to waiting for reward. In intolerance-to-delay (ID) protocols, animals must choose between small/soon (SS) versus large/late (LL) rewards. In the probabilistic discount (PD) protocols, animals are faced with choice between small/sure (SS) versus large/luck-linked (LLL) rewards. It has been suggested that PD protocols also measure impulsivity, however, a clear dissociation has been reported between delay and probability discounting.

**Results:**

Wistar adolescent rats (30- to 46-day-old) were tested using either protocol in drug-free state. In the ID protocol, animals showed a marked shift from LL to SS reward when delay increased, and this despite adverse consequences on the total amount of food obtained. In the PD protocol, animals developed a stable preference for LLL reward, and maintained it even when SS and LLL options were predicted and demonstrated to become indifferent. We demonstrate a clear dissociation between these two protocols. In the ID task, the aversion to delay was anti-economical and reflected impulsivity. In the PD task, preference for large reward was maintained despite its uncertain delivery, suggesting a strong attraction for unitary rewards of great magnitude.

**Conclusion:**

Uncertain delivery generated no aversion, when compared to delays producing an equivalent level of large-reward rarefaction. The PD task is suggested not to reflect impulsive behavior, and to generate patterns of choice that rather resemble the features of gambling. In summary, present data do indicate the need to interpret choice behavior in ID and PD protocols differently.

## Background

Lack of self-control abilities is an important symptom of many psychiatric disorders, notably in the attention deficit/hyperactivity disorder (Sagvolden and Sergeant, 1998; Swanson et al., 1998; Sonuga-Barke, 2003). There is indeed a growing interest in the study of impulsive decision in humans (Krawczyk, 2002; Bechara, 2004). As for animal models, many different aspects of impulsivity have been studied with operant-behavior paradigms. In laboratory settings, impulsive behavior can be defined in terms of "poor" decision making, based on anomalous processing of actual incentive values of the two alternatives (Evenden, 1999; Ho et al., 1999; Monterosso and Ainslie, 1999). In one of the most widely adopted paradigms, the intolerance-to-delay (ID) protocol, slightly food-restricted animals are tested in operant-behavior cages, where they are provided with choice between either one immediate pellet of food (small-and-soon, SS) or five pellets of food after a delay (large-but-late, LL). Animals classically shift their preference from the LL to the SS reward as delay increases. Since impulsive subjects are intolerant to the forced waiting for a delayed reward, a flatter or a steeper shift towards SS choice are a classical index of reduced or increased impulsivity, respectively (Evenden and Ryan, 1996, 1999).

It has been suggested that impulsive subjects may also avoid conditions where the reward income is made uncertain. Such suggestion implies that both "uncertainty" and delay of reward will elicit the same underlying form of intolerance (Mazur 1995; Rachlin et al. 1991). This is based on the assumption that any randomly omitted reward will force animals to wait for one of the next trials (the "lucky" one) to get actual reinforcement, and that they need to cope with this unpleasant but forced procrastination. It has been demonstrated that the perceived value of a given reinforcer is discounted similarly following both "delay" and "probabilistic gaps" in the delivery of rewards (Richards et al., 1999; Ho et al., 1999). Moreover, the detection of reward value across its delayed or uncertain delivery has been suggested to require a common neural substrate, namely an intact nucleus accumbens (Cardinal and Cheung 2005; Cardinal and Howes 2005). However, there is evidence that time- and probability-induced discounting are different and dissociable processes (Green et al. 1999; Green and Myerson 2004). This notion should be kept in mind, since a shift from a small-and-sure (SS) towards a probabilistic (large-but-luck-linked, LLL) reward is often discussed in terms of impulsivity-driven aversion for uncertainty in classical literature studies that used probabilistic-discount (PD) protocols (Mobini et al., 2000, 2002).

A crucial point in intolerance-based tasks is to consider whether the actual parameters make the shift in preference (from large reward in either protocol, termed LL/L, to SS) economically convenient or not. In the ID task, delay duration is always imposed in a range of values which render any SS shift overtly sub-optimal, and this assumption is actually confirmed by present data. Conversely, within uncertainty-based tasks, probability ("p") values to be imposed can be divided into two distinct fields, separated by the indifference-point "p" value (calculated as "small reward size"/"large reward size", e.g. 20% in our present work, where SS was 1 and LL/L was 5). In the range of "p" values before the mathematical indifference point (100% > p > 20%), the risk of large reinforcement loss is mild relative to its size. Under these conditions, it is still "economically" convenient for rats to choose the LLL (average outcome being still more than one pellet per nose-poking) over SS reward (only one pellet per nose-poking). Hence, if animals display a shift towards SS, this would be interpreted as an intolerance reaction against uncertainty. In the range of "p" values beyond the mathematical indifference point (20% > p > 0%), it becomes "economically" convenient for rats to choose SS (one certain pellet per nose-poking) over LLL reward (the average outcome being less than one pellet per nose-poking). In classical literature studies (Mobini et al., 2000, 2002), untreated control animals showed a shift from the probabilistic towards the certain reward, but this happened under conditions that rendered such a shifting actually optimal in terms of food gain. This consideration raises the possibility that the observed shift rather represented the obvious consequence of the natural drive towards food maximization. By definition, impulsive decisions are taken "without consideration of (negative) consequences". Hence, only those behavioral responses, which can be demonstrated to be still adopted despite adverse consequences on total foraging, can be defined as truly impulsive.

In the present experiment, we wished to directly compare the ID and PD protocols, to evaluate whether or not adolescent rats would display an equivalent intolerance towards delay vs uncertainty of reward delivery. The specific age-period for testing was chosen mainly because the peculiar behavioral instability, which is typical of adolescence as demonstrated by previous work (Laviola et al., 1999, 2003; Spear, 2000), might be pushing animals towards extreme drives at this age, such as novelty- and risk-oriented behavior. We employed a range of delay/probability values under which the "optimal" choice would always be the large reward. In this way, any shift towards the SS reward would be a "true" index of impulsivity-driven intolerance, since it would be anti-economical from the point of view of foraging optimization. As expected, in the ID protocol, animals shifted towards the SS reward, a classical index of impulsive choice. Conversely, in the PD protocol, animals did not shift against economical convenience. Rather, their preference for the LLL reward was very stable, being maintained even when its delivery was made very infrequent, so that it would be economically convenient to shift to the SS one, at least in theory.

As a final remark, we wish to underline that the indifference point is separating, in both protocols, the field where LL/L choice is always optimal from the field where the SS choice would be optimal. Such turning point (i.e. odds against obtaining the large reinforcer being 4 in our experiment) is mainly based on abstract mathematical calculation and comparison of the final payoff obtained with a clear-cut (i.e. 100%) SS vs LL/L choice. However, the behavioral strategy adopted by rats is rather complex, consisting of preferential choice at a given hole plus a lower but constant patrolling at the other hole. Under these conditions, rats are unlikely to detect very slight differences between the two alternative outcomes, and may hence be under quasi-indifference conditions. Thus, rather than being pushed by overt economical convenience, animals might express their hole-preference based on the emotional evaluation of rewarding features issuing from either alternative option. A further aim of the present work was to compare the economical contingencies of the two procedures around the indifference point. Specifically, we compared actual foraging with theoretical food gain, ideally obtained by maintenance of LL choice in ID task and by a shift to SS reward in PD task. Specifically, we calculated what amount of food rats could have eaten if they behaved just as their siblings in the other protocol did. Such a calculation allowed to assess what economical conditions rats were actually facing. Such analysis was focused on the specific window around the mathematical indifference point, which represents a sort of upper limit in intolerance-based tasks. Indeed, to avoid the bias of an economically-driven preference shift as outlined above, task contingencies shall be able to generate phenomena of behavioral intolerance by this point.

## Results

### Choice behavior in either protocol

Following one week of training, all rats exhibited as expected a significant preference for the large over the small reward. However, a certain "baseline" level of nose-poking for small reward (average choice: 30.5 ± 4.4% for the small and 69.5 ± 4.4% for the large reward) was always present. This finding replicated previous experiments in our lab (Adriani et al., 2004), indicating that animals were never completely attracted by the larger reward, but constantly probed the outcome of nose-poking at the other hole.

When the delay or the probabilistic challenge were gradually increased over days (see Table 1), interestingly the profile differed as a function of the task, protocol × session, F(10,70) = 8.96, p < .001 (see Figures 2 and 3). Namely, in the ID protocol, rats showed a dramatic shift towards SS choice at the highest delays. This profile is classically described as a manifestation of intolerance, generated by the delay. Indeed, by definition, the optimal choice would be to maintain nose-poking for large reward, at least until a delay of 100 s, which would be substantially equivalent to odds = 4 (the mathematical indifference point, see Table 1). The shift towards SS reward seems a nice form of impulsive decision making, with adverse consequences on total foraging. Conversely, in the PD protocol, rats showed quite the opposite profile. Namely, a robust trend was shown towards enhanced preference for the LLL choice, which attained a maximal level of 87.5 ± 5.1%. With the PD protocol, we never observed a shift towards SS choice. Data indicate no such a reaction, even in spite of a high degree of probabilistic rarefaction (odds 5 and 6).

This profile may appear somewhat "surprising", based on the classical prediction of uncertainty-induced reward discounting. However, present data are a clear-cut replication of those recently obtained by our group, showing the development of LLL preference in control adolescent rats (Adriani et al., 2005). To explain why discounting of the uncertain reward does not occur, we suggest a possibe role for two key parameters adopted in these two works: 1) a fivefold magnitude of the large versus the small reinforcer, rendering the former highly attractive, and 2) the adoption of a gradual day-by-day increase of uncertainty levels, which possibly allowed rats to adapt progressively to such a challenge. In these conditions, animals preferred to wait for an "extremely lucky" event, being perhaps attracted by "binge" reinforcement, without showing any intolerance to its progressive rarefaction, possibly because the rise of uncertainty was gradual.

### Economic features of the two protocols

At the indifference point, an approximated and simplified calculation about the maximal number of trials, available in the time-limited session, shows that 1500 s (25 min) can leave room to 75 trials × 20 s (1 s + 15 s timeout + 4 s spontaneous waiting) in the PD task (and also in the ID task with 100% SS selection), or to 18/19 trials × 80 s (1 s + 60 s delay + 15 s timeout + 4 s spontaneous waiting) in the ID task with 100% LL selection. These two approximated values represent the two extremes of a range where actual figures can lay. The average number of trials (LL/L+SS) actually completed by rats was 59.0 ± 2.2 in the PD task, and 22.6 ± 1.4 in the ID task. The mean inter-trial interval actually shown by rats, namely the spontaneous waiting between the end of a timeout interval and the next nose-poking choice, was 9.9 ± 1.3 s in the PD task and 22.6 ± 3.3 s in the ID task, as calculated from raw data generated by animals. These results suggest that ID rats were somewhat slower in completing a novel trial than their PD siblings.

In the PD task, where no delay was present, the only time-constraint was represented by the 1 s-interval between nose-poking and food delivery plus the 15 s-timeout. Animals were thus free to express under availability of a maximal number of trials. For the ID task, each LL choice triggered a very long delay, which represented an inflexible obstacle against expression of further food-rewarded nose-poking acts. Two hypotheses can be raised: first, the delay constraint during the ID task might have increased the waiting abilities, possibly reducing the willingness of rats to express quick nose-poking. Second, the stochastic reward omission might be acting as a stimulating factor within the PD task, motivating animals to further nose-poke soon after a timeout had elapsed.

The core assumption of ID testing is that a shift towards SS is sub-optimal and should be avoided. This was confirmed by theoretical data, generated under the hypothesis that rats in the ID task maintained a preference for LL, like their PD siblings did (see Table 2). In this case, as revealed by a highly significant main effect of payoff, F(1,7) = 27.7, p < .001, a significant difference in total food gain was evident between "actual" (shifting towards SS) and "potential" (non-shifting, LL preferring) choice behavior. Specifically, the LL option rendered significantly more food than the SS one, clearly indicating that actual testing conditions were well before the indifference point. Despite a priori calculations, which set a delay = 60 s in correspondence to the indifference point, a posteriori data on equivalence between odds/probability and delay demonstrate that the actual indifference point was expected around delay = 100 s (Table 1). Thus, in the ID task, rats appear to be under conditions where economical convenience loaded onto the LL option, yet a strong delay-induced LL aversion was observed. Since the latter was expressed despite wide anti-economical consequences, we can conclude that it can reliably be used as a valid index of impulsivity.

Another interesting set of figures is generated by assuming that rats in the PD task showed a shift towards SS like that shown by their ID siblings (see Table 2). In this case, as revealed by the lack of significance for the payoff factor, F(1,7) = 0.399, NS, no significant differences in total food gain were evident between "actual" (non-shifting, LLL preferring) and "potential" (shifting towards SS) choice behavior. Hence, rats appear to be under conditions of economical indifference, as it was expected at least for odds = 4. As for odds of 3 and 5, the difference in food gain between "actual" and "potential" behavior is slight and non significant, so that rats appear to be under a condition of substantial quasi-indifference. Interestingly, a strong LLL preference is always expressed across all odds values. These data suggest that uncertainty factors were not strong enough to produce any consequences, despite the definition of impulsivity would require uncertainty to produce an anti-economical intolerance, as was the case in the ID task. It is likely that further increase in odds value would eventually generate a SS shift, but such a shift would then be promoted also by economic convenience, thus questioning the validity of this parameter as a measure for impulsivity.

## Discussion

This study compared the behavioral reaction to rarefaction of large reinforcement in a pair of similar two-choice operant-behavior tasks, where preference for large or small rewards is assessed in food-restricted rats. We wish to underline that the present animals were trained to consume their daily meal during the testing session and shortly when placed back into the home cage. In this way, they were facing a 23 h/day feeding absence, and were hence motivated to work for food during operant testing. While undergoing this procedure, which is largely adopted in the literature, rats are not under starvation nor malnutrition, since they fully express a normal behavioral repertoire in the home cage, including a playful social behavior. The attributes of food reward in either test share some similarities and some key differences. Namely, the small reward (SS) is always for sure and comes immediately, whereas the large reward delivery may be either delayed (ID protocol) or uncertain (PD protocol). Specific values, to be run for each daily session, were selected after an a priori calculation of the correspondence between delays and odds/probability, aimed to produce a similar level of rarefaction for LL/L delivery in both protocols. This was to be sure that the striking differences in behavior, generated by the two protocols, could not be explained by gross differences in the maximal frequency of large rewards potentially obtained, and should be ascribed to other intrinsic key features. An a posteriori re-estimation of equivalence between odds/probability and delays was conducted, which scaled the whole curve generated by the ID task towards the left. As a result, differences between choice profiles in the two tasks were even strengthened. Indeed, testing of animals until a delay of 150 s within the ID task, which would be substantially equivalent to odds = 6 reached in the PF task, likely would produce a further enhancement of the dissociation observed between the two profiles.

The experimental observations reported here demonstrate that the profile shown in PD protocols may differ substantially from classical intolerance/aversion observed in ID protocols. As classically reported, animals in the ID protocol then developed a robust aversion for the longest delays, and showed a marked shift from LL to SS choice. At this point, animals are likely to have realized that there is nothing to do to avoid the elapsing of delay intervals, and are therefore likely to develop an anti-economical intolerance (Evenden, 1999; Ho et al., 1999; Monterosso and Ainslie, 1999). Conversely, animals tested in the PD protocol did not show the same profile. As a reaction to an increasing proportion of omitted reinforcement, animals developed and maintained a robust preference for LLL reward. Such behavior may in part be explained by the fact that chamber and magazine lights, required to signal starting and ending of the time-out period, were also turned on when food delivery was omitted. These lights may indeed be acting as a conditioned reinforcers, and sustain LLL-seeking behavior until the primary reinforcer eventually comes (as in second-order schedules, see Everitt and Robbins, 2000). These considerations may help formulating a possible explanation, namely that training with the uncertainty challenge was gradual enough for rats to realize that large reinforcement eventually comes, despite the repeated and unpredictable omissions. Preference for LLL until odds of 5 and 6 is striking, since over 80% of these nose-poking demands did not trigger any food delivery. It is intriguing to note that the average waiting-time for the eventual LLL reward was 60 seconds or more, a delay which produced a considerable aversion for LL reward when assessed in the ID protocol.

It is noteworthy that animals faced with either delays or omissions reacted in two distinct ways. After a substantial similarity at the mildest levels of LL/L rarefaction, choice curves began to diverge beyond odds = 1, which was substantially equivalent to delay = 30 s. In our opinion, the crucial difference between LL and LLL rewards is that the former is delayed in a signaled and predictable manner, whereas delay in obtaining the latter is completely unpredictable and luck-linked (Cardinal et al., 2000). Indeed, once triggered, the delay in the ID protocol must follow its scheduled duration, and there is nothing that animals can do. This situation is likely to induce a state of frustration, thus generating intolerance/aversion for delays. Conversely, after each omitted large-reward delivery in the PD protocol, animals have the possibility to express another nose-poking choice. In general, there are more opportunities of nose-poking choices in the PD protocol, and this may help animals to feel that the situation is more "under control". A great number of studies have shown that controllability of stress sources helps animals to cope with adverse contingencies, and hence uncertainty of LLL reward should be not necessarily expected to produce aversion.

From our data, it seems that LLL rewards are actively preferred by rats. Animals apparently adopted the strategy to wait for a "lucky" but "rare" event, rather than collecting many smaller reinforcement. There are two possible explanations for this finding. One is that animals developed a "habit", the other is that rats were perhaps attracted by "binge" reinforcement, without being affected by its uncertainty neither by its rarefaction. As for the first possible explanation, the finding of minor effects on choice behavior in the PD protocol may indicate that the choice of nose-poking hole somewhat continued to occur independently from the outcome. This could suggest that rats developed a compulsive and perseverant hole preference, possibly due to the establishment of a behavioral habit. In other words, normal rats seem to express a sort of fixed habit-based responding, rather than being open to an evaluation of the actual outcome. Accordingly, recent findings (Adriani et al., 2004) raise the question of behavioral inflexibility (with a tendency to perseveration) shown during preference shifts, and suggest that some individuals may be less flexible than others (Evenden, 2002; Laviola et al., 2003). However, whilst PD rats were quite insensitive to LLL rarefaction, their ID-protocol siblings quickly displayed intolerance against large-reinforcement delay. ID subjects showed a shift to SS choice, a finding fully consistent with impulsivity-based responding, according to previous work (Evenden and Ryan 1996, 1999). It seems unlikely that PD rats were characterized by a lack of flexibility when their ID siblings were able to shift in their choice. Thus, we propose that the PD protocol generates a true "instinctive" preference for LLL rewards. Noteworthy, our present findings unequivocally suggest that rats prefer a random-coming reward, delivered eventually and all-at-once, rather than a similar overall gain, collected by slow accumulation of smaller unitary bits. In PD rats, the salient cue to decision seems to reside into the size attractivity of unitary reward, and not in frequency of its delivery nor in the total amount gained (or lost) over time.

Classical definitions of impulsivity predict that a given factor (delay, uncertainty) shall discount large-reward value, and that a choice shift is then observed against economic convenience of the outcome (Evenden, 1999; Ho et al., 1999; Monterosso and Ainslie, 1999). The phenomenon of uncertainty-produced discounting did not occur in our hands. It is likely that animals would eventually show a shift towards SS, at least for "p" values much lower than those presently tested. However, a shift to SS reward would be economically convenient in these conditions, and cannot therefore be used as an index of impulsive behavior. Indeed, only anti-economical choices can be considered an unbiased and reliable index in the field of impulsive decision making (Evenden, 1999; Ho et al., 1999; Monterosso and Ainslie, 1999). Such kind of considerations also apply to interpretation of data by Mobini and colleagues (2000, 2002). These authors employed a "small"/"large" reward ratio of 0.5 (Mathematical indifference point at "p" = 50%) and tested rats under a range of "p" values well beyond this point (Mobini et al., 2000, 2002). In our opinion, by promoting a preference shift according to (rather than against) economical convenience, these features do bias the preference shift as a parameter for impulsive decision making. Abnormalities in the process of large-reward discounting, observed by these authors in rats after various manipulations, might well be discussed in terms of altered perception of reward magnitudes and/or deficits in comparing global payoff of either choice option. In other words, their protocol contingencies may be useful to evaluate abnormalities in the emergence of an economically-forced shift in choice behavior, being conversely not suitable to measure impulsive choice, by definition.

Two considerations must be highlighted here regarding models of reward discounting. It is assumed that, when animals discount the value of a delayed or uncertain reward, then the value of the SS reward is compared against the discounted value of the LL/L reward to take a decision. These considerations are put forward by also assuming that perception of reward size is equivalent in both paradigms and that odds against rewarding may be compared across the two protocols. A first consideration is that delay-induced discounting is apparently a more reliable phenomenon than uncertainty-induced discounting. In other words, rats poorly cope with delay and inescapable waiting constraints, whereas they accept unpredictable omissions in reward-delivery. Moreover, under the present conditions, where the final net foraging was not affected, rats preferred to work for "binge wins", even at high levels of rarefaction, rather than shifting towards a SS-seeking strategy. The latter, made of nose-poking for a lower and constant outcome, would imply a flatter distribution of food gains. Such observation again suggests that very similar temporal distributions, made of bouts of five pellets coming far apart each other, are potentially generated in both tasks. However, such kind of distribution can be avoided, when the distance between bouts is an inflexible constraint (ID task), and can conversely be preferred, when its length varies in an unpredictable way (PD task). One possible explanation for the preference, displayed in the latter task, is that the LLL-choice behaviour may be reinforced by the contrast between many no-food trials and the eventual five-pellet one. Such kind of contrasts have been shown to increase dopaminergic activity in the midbrain (Fiorillo et al., 2003), and may support risk-taking behaviour (Van den Bos et al., 2005).

A second consideration is that there might be a pitfall in those models, where criteria for decision reside in comparison between unitary value of SS versus unitary discounted value of LL/L reward. This assumption may stand valid for a sort of "instinctive" decision process, where two alternative unitary values are first discounted and then compared. But more "evolved" decision processes may occur, where animals may be capable to take into account the global convenience of a choice strategy, by considering delivery failures and food amount earned (or lost) across multiple choices. This kind of process requires the ability to consider, beyond each single choice, the estimated quantity of food gained having access to many choices during a given time interval. Without taking into account this process, the conceptual models describing decision making may be limited. In this light, the PD task adopted here is suggested to unveil attraction for "binge and rare" over "low and constant" reinforcement, when rarefaction is obtained by random omissions (rather than a constant delay) of reinforcer delivery. This behavioral drive, consisting of willingness to seek for a highly rewarding sensation despite association with some negative features (such as random rarefaction in the present case), is not surprising. Much effort has been devoted to study determinants of risk-seeking drives, in terms of neurobiological substrates and adaptive function (Bardo et al., 1996; Laviola et al., 1999). Present observations may open new perspectives for studies in the field of peculiar (patho)physiological conditions, such as sensation seeking, reckless behavior, and gambling.

## Conclusion

There is no doubt that behavioral output in a two-choice operant paradigm provides precious information about decision-making strategies. However, we showed quite clearly that the decision taken by animals, in terms of choice between a larger (but less frequent) versus a smaller reinforcer, provides insights that do not necessarily reside within the "impulsivity" dimension. Depending on protocol contingencies, and specifically in the absence of the strong aversion otherwise elicited by inflexible delays, other dimensions emerge, such as the natural attraction for "binge" reinforcement. Under conditions of economical quasi-indifference, i.e. when there were no overt adverse consequences on the total foraging, such attraction was able to sustain the LLL preference, despite a considerable rarefaction in actual food delivery. Under these contingencies, animals can freely choose a temporal profile of reward distribution, ranging between two possible extremes: a "sharper" one, made of random-coming "lucky-strike" episodes, separated by time intervals of uncertain duration, or a "smoother" one, obtained by a more frequent alternation of either choice. The latter strategy would possibly allow to dilute the frustration, associated with the unlucky attempts to obtain more food, with a more frequent nose-poking for the single and certain pellet. However, not always laboratory animals are driven by simplistic cost/benefit rules. It is known that some strategies, being apparently less rewarded and/or requiring more effort, are often adopted and perhaps more adaptive, a phenomenon called "contrafreeloading" (Inglis et al., 1997). In the present specific case, rats may be specifically motivated by the "temptation to gamble" and may conversely find more "boring" a monotonous collection of small bits. In this frame, we propose that such a behavioral dimension might more closely resemble features that are typical of a "gambling" trait. The present study may thus provide novel useful insights for the interpretation of PD tasks.

It should be underlined that these two protocols may be used to probe animals for the balance between "primordial" and "evolved" drives. In other words, a similar process is likely to be required to overcome aversion to delay and attraction for "binge" rewarding, in order to produce more self-controlled individuals. In theory, it should be possible to modulate the weight of primordial "delay aversion" and "binge-reward attraction" drives, and/or the ability to inhibit these primordial drives to express an evolved and truly self-controlled response. Such ability is known to require intact serotonergic activity (Wogar et al., 1993; Harrison et al., 1997; Puumala and Sirvio, 1998; Dalley et al., 2002), expecially within the pre-frontal cortex (McClure et al., 2004; Ridderinkhof et al., 2004) and the cortico-striatal projections (Cardinal et al., 2004; Christakou et al., 2004). We have recently obtained some data, showing that exposure to methylphenidate during adolescence resulted in enduring changes, adult rats being more self-controlled in both tasks. Specifically, methylphenidate-exposed rats were less impulsive in the ID protocol (Adriani et al., in preparation), thus increasing their overall food gain, and showed an increase of low-risk certain-payoff nose-poking in the PD protocol, thus smoothing the temporal distribution of foraging and diluting the uncertainly-rewarded periods (Adriani et al., 2005). These animals were apparently not driven by "delay-avoidance" nor by "binge-attraction". Rather, they seemed to overcome such instinctive drives, by looking beyond single-choice outcome and by taking into account the long-term payoff.

A final remark shall underline that the present data were obtained on adolescent rats, which are known to differ substantially from adults in reward-related manifestations (Spear, 2000; Laviola et al., 1999, 2003). Whether these findings can be extended to adult rats remains an open question, deserving further work. We demonstrated previously that adolescent mice are more impulsive than adult ones (Adriani and Laviola, 2003) but, to the best of our knowledge, there are no rat studies comparing age-dependent profiles for either ID or PD task. Present data are however relevant in view of recent literature, suggesting that animal models of adolescence may be useful to evaluate age-related physiological trajectories that may, in some cases, progress into psycho-pathological processes (Andersen, 2003). In this frame, elaboration of correct expectations from two-choice protocols is essential for a deeper psycho-biological investigation of reward-seeking abnormalities.

## Methods

Experimental protocols were approved by institutional authorities and are in close agreement with European Community Directives and with the Italian Law. All efforts were made to minimize animal suffering, to reduce the number of animals used, and to use alternatives to *in vivo *testing.

### Subjects, and rearing conditions

Eight Wistar pregnant female rats (Harlan, Italy) were housed in an air-conditioned room (temperature 21 ± 1°C, relative humidity 60 ± 10%), with a 12-hr light-dark cycle (lights on at 8.00 am). Water and food (Enriched Standard Diet, Mucedola, Settimo Milanese, Italy) were available *ad libitum*. The day of delivery was considered as post natal day (PND) zero, pups being culled to 6 males and 2 females. Pups were then weaned on PND 21 and housed in groups of two siblings, according to sex. Only two male subjects per litter were used in this experiment, the other four male subjects being used for other experiments. Within each litter, one sibling was assigned to the ID protocol group and the other to the PD protocol group (n = 8). Animals were tested for choice behavior in a drug-free state during adolescence (PND 30 to 46).

### Two-choice operant-behavior tests

Animals were tested in one of two protocols, involving a delay- or probability-based rarefaction of large reward (see Introduction). Before the schedule started, animals were food-restricted for two days, to keep them at 80–85% of their free-feeding weight in order to increase their motivation to work for food delivery. Each animal was then placed daily in a computer-controlled operant chamber (Coulbourn Instruments, USA), provided with two nose-poking holes, a chamber light, a feeder device, a magazine where pellets (45 mg, BioServ, USA) were dropped, and a magazine light. The nose-poking in either hole was detected by a photocell and was recorded by a computer, which also controlled food delivery. After the 25-min session, animals were returned to their home cage, where they were given standard chow (approximately 10 g/each). During the daily sessions, rats were able to eat approximately 3 g of food, i.e. only a small part of their daily need. It is therefore unlikely that levels of hunger experienced by rats changed substantially during the session. This methodological remark is important, since self-control measures are known to be directly modulated by levels of hunger (Kirk and Logue, 1997).

During the training phase (one week), nose-poking in one of the two holes resulted in the delivery of five pellets of food, whereas nose-poking in the other hole resulted in the delivery of one pellet of food. After nose-poking and before food delivery, the chamber light was turned on for 1 second. Following food delivery, the magazine light was turned on for 15 seconds, during which nose-poking was recorded but was without scheduled consequences (time-out). The magazine light was then turned off, and the system was set ready for the next food-rewarded nose-poking trial. The total number of trials and the inter-trial time were not fixed, since rats were free to express nose-poking for food at their individually-variable rate during the 25-min session.

During the testing phase (one week), the two protocols differed slightly. In the ID protocol, a signaled delay was added to the 1 s-interval, normally scheduled between nose-poking and large-reward delivery. The chamber light was kept on during the entire length of this delay. The small reward delivery was unchanged. Hence, animals had choice between a "LARGE & LATE" (LL) or a "SMALL & SOON" (SS) reward. In the PD protocol, a probabilistic dimension was associated to the delivery of the large reward. The chamber and the magazine lights were turned on after nose-poking following the usual schedule. However, sometimes the delivery of large reward could be omitted, according to a given level of probability ("p" = percentage of actual food delivery over total demands). The small reward delivery was unchanged. Hence, animals had choice between a "LARGE & LUCK-LINKED" (LLL) or a "SMALL & SURE" (SS) reward.

**Figure 1:**
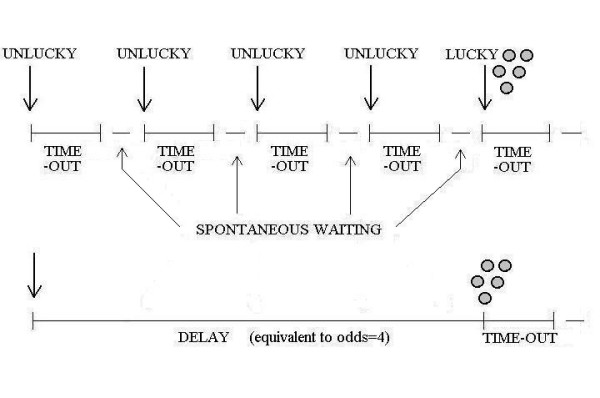
Schematic diagram showing equivalence between ID and PD protocols at the mathematical indifference point (odds against reinforcing = 4). Arrows represent nose-poking for large reward by rats. In the PD protocol, there are an average of four "unlucky" events (omissions) before a "lucky" event (5-pellet food delivery). In the ID protocol, the delay interval should be at least four times the time-out (delay = 15 × 4 = 60 seconds) to reproduce a 5-pellet delivery with approximately the same temporal characteristics as in the PD protocol. To turn this ideal situation into a more realistic account, four times the spontaneous waiting *observed *between two consecutive trials (further 10 × 4 = 40 seconds) shall be *added *to delay duration (see Table 1). To optimize performance for long-term benefit, choice should be LL/L before the mathematical indifference point, and SS beyond it.

**Figure 2:**
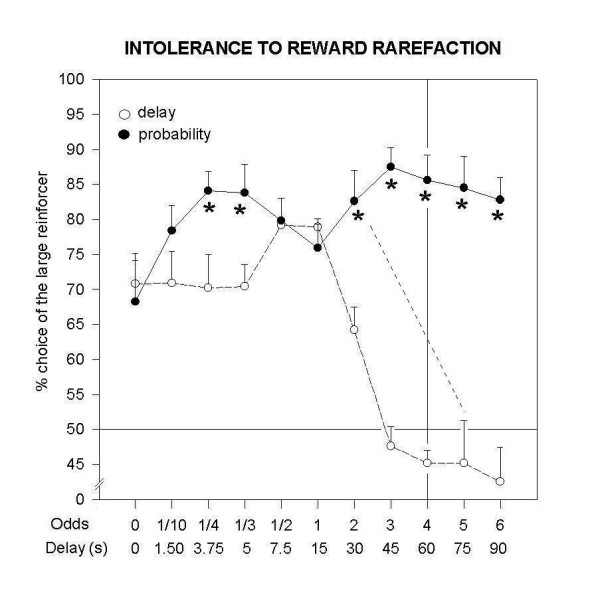
Choice behavior in rats (n = 8) tested with either the intolerance-to-delay (ID) or the probability discounting (PD) protocols, shown during subsequent daily sessions. Data represent the mean (± SEM) choice (%) of the larger reinforcer, delivered either after a delay (LL in the ID protocol) or with a certain degree of probability (LLL in the PD protocol). In correspondence to each odds level, delay duration to be run was calculated a priori (see also Table 1). Equivalence between odds/probability and delay was re-estimated a posteriori (see e.g. dotted line). The vertical line shows the mathematical indifference point in the PD task, separating the field before (left to the line) from beyond (right to the line) it. The horizontal line shows the level of rats' indifferent choice, separating preference for LL/L (above the line) from preference for SS (below the line). * p < 0.05 when comparing across protocols within a given daily session.

**Figure 3:**
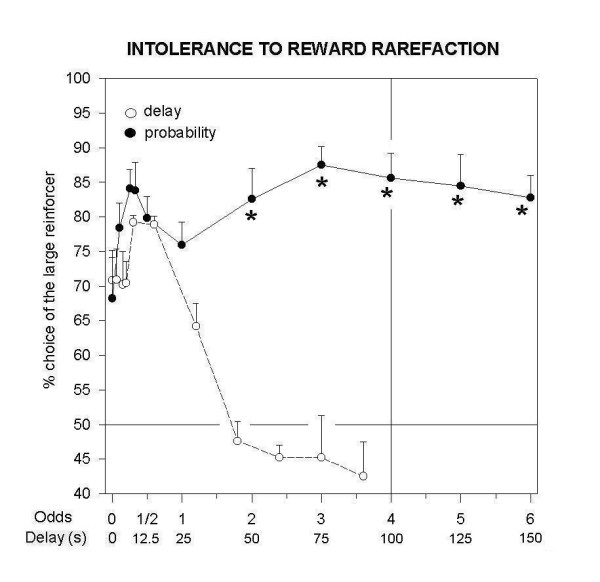
Choice behavior in rats (n = 8) tested with either ID or PD protocols. Data are the same as shown in Figure 2, but the ID-task curve has been scaled according to the a posteriori re-estimation of equivalence between odds/probability and delay (see also Table 1). The asterisks denote daily sessions at which points of the PD tasks were significantly different from the corresponding points of the ID task. It is clearly evident that the delay dimension produced *aversion *for LL, whereas the probabilistic dimension produced a *preference *for LLL.

The delay length and the probability level were kept fixed for each daily session, and were changed progressively over days (see Table 1). Only one session was run for each delay value and "p" level, which were thus changed daily. To be sure that data generated in both protocols were comparable, delay and probability values were chosen in order to produce a similar rarefaction level in actual delivery of large reward in either protocol (termed LL/L). In other words, within each given daily session, the number of large rewards earned per time unit had to be similar in both protocols, as far as possible. This was obtained by referring probability values to the corresponding "odds against reinforcing", which are defined as the mean number of omitted large-reward delivery ("unlucky" events) before an actual delivery ("lucky" event). The relation between probability values and odds is simply mathematical, the formula being "p" = 1/(odds+1). When moving from the PD to the ID protocol, the delay to be imposed (for ID task) corresponds to the length of time elapsed during timeouts of unlucky events (for PD task). It is easy to demonstrate then that delay = timeout × odds (see Figure 1). This is an ideal and limit situation, in which the incidence of small-reward choices, and of inter-trial intervals generated by spontaneous waiting, is obviously not taken into account. A more realistic account would require that delay = (timeout + inter-trial) × odds. It is substantially evident that any odds-equivalent delay duration is actually longer in the realistic than in the ideal situation. However, since the inter-trial value cannot in any way be predicted a priori, the delay duration to be run for each daily session, in correspondence to each odds level, was calculated before the experiment in the ideal situation (no spontaneous waiting). Equivalence between odds/probability and delay was re-estimated a posteriori, by considering spontaneous waiting (around 10 s in the PD task, see Results). Table 1 shows the delay duration chosen for testing (a priori values), and odds-equivalent delay values, re-estimated after the experiment (a posteriori values). In other words, the white point at delay = 75 s was substantially equivalent to the black point at odds = 3 (see Figure 2, dotted line). Hence, the curve generated in the ID task was scaled to the left, to an extent depending on the re-calculated equivalence (see Figure 3).

The mathematical indifference point, at which either choice was mathematically identical in terms of total foraging, was odds = 4 ("p" = 20%), corresponding to delay = 60 s (at least from a priori calculations in the ideal situation). We imposed a range of delay and "p" values before the mathematical indifference point, when LL/L was always the optimal choice (see Introduction). Rats were also tested around the mathematical indifference point, when none of the options was economically optimal (see Table 2). Rats were not tested far beyond the indifference point.

### Economic features of the two protocols

In both protocols, around the mathematical indifference point (i.e. sessions with odds 3 to 5), we calculated the number of food pellets actually earned by rats. We also calculated the potential number of pellets that each rat could have gained with an opposite choice-preference behavior. This was done by assigning, to each individual rat, the percent choice value actually expressed by its sibling. Specifically, the total number of trials actually completed by each rat during the session (LL/L+SS nose-poking) was re-assigned between LL/L and SS, based on percent choice shown by the sibling rat in the other protocol. For the ID protocol, the total number of trials (LL+SS) was also facing an upper limitation, due to the time-limited session. In particular, the amount of time needed to express the novel estimated number of trials (re-assigned LL + re-assigned SS) was calculated, in terms of delay duration plus timeout intervals for each rat. In one case, this sum exceeded the total session duration (25 min), and re-assigned LL and re-assigned SS values were thus diminished proportionally. The total number of theoretically potential pellets was always calculated as 5*re-assigned-LL + re-assigned-SS. For the PD protocol, we first calculated the re-assigned SS and LLL trials by assigning to the PD rats the percent choice of the ID sibling. Once estimated, the number of trials with a LLL attempt was then turned into "lucky" LLL trials based on the level of "p" actually experienced by the PD rat itself. The number of theoretically potential pellets was 5*re-assigned-LLL*actual-p-value + re-assigned-SS.

### Design and data analysis

Data were analyzed by randomized-block ANOVA. The first dependent variable was the choice (%) for the large reinforcer, namely percentage of LL/L over total LL/L+SS choices. The general design of the experiment was a 2-level protocol (ID vs PD) × 11-level session ("p" or delay level fixed for each session) factors. The dependent variable of the second analysis was the total number of pellet, in order to compare those actually eaten with the potential food gain, calculated theoretically as described above. Data from ID and PD tasks were analyzed separately. The design was a 2-level payoff (actual vs potential) × 3-level session (odds level: 3 to 5) factors. Multiple comparisons were performed with the Tukey HSD Test.

## Abbreviations

(ID) intolerance to delay

(PD) probabilistic discount

(SS) small/soon or small/sure

(LL) large/late

(LLL) large/luck-linked

(PND) postnatal day

## Competing interests

The author(s) declare that they have no competing interests.

## Authors' contributions

WA proposed the experimental design and carried out all the experimental work.

GL is the principal investigator of the research project that supported this study.

## Figures and Tables

**Table 1:** Synoptic table of comparison among odds, "p", and delay values across ID and PD tasks.

Odds	"p" (%)	A priori Delay (s)	A posteriori Delay (s)	
0	100	0	0	
1/10	90	1.50	2.50	
1/4	80	3.75	6.25	
1/3	75	5.00	8.33	
1/2	66	7.50	12.50	
1	50	15	25	
2	33	30	50	
3	25	45	75	

4	20	60	100	Indifference Point, PD task

5	16.6	75	125	
6	14.3	90	150	

Delay duration corresponding to each odds level was calculated a priori in the ideal situation (no spontaneous waiting). Equivalence between odds/probability and delay was then re-estimated a posteriori for a more realistic account (the mean inter-trial interval due to spontaneous waiting was around 10 s in the PD task).

**Table 2:** Number of food pellets received in either task around the ideal/mathematical indifference point, compared with the "theoretical" food gain (amount of food pellets that rats could have eaten if they behaved just as their siblings in the other protocol did, n = 8).

		ID task			PD task		
Delay (s)	Odds	Actual SS shift		Potential LL preference	Actual LL preference		Potential SS shift

45	3	83.3 ± 2.5	vs	123.3 ± 5.9 *	69.0 ± 6.4	vs	61.0 ± 5.5
60	4	67.9 ± 1.6	vs	100.8 ± 6.6 *	54.5 ± 3.8	vs	55.8 ± 4.3
75	5	58.3 ± 1.8	vs	84.2 ± 7.9 *	43.8 ± 3.6	vs	47.8 ± 3.3

In the ID task, it would be economically convenient to keep preferring the LL choice, demonstrating that task contingencies are still before the indifference. In the PD task, either choice is substantially indifferent from the economical viewpoint, demonstrating that task contingencies are under conditions of quasi-indifference. * p < 0.05 in post-hoc comparisons drawn between the "actual" and the "potential" food amounts.
